# F33. MATERNAL AND PATERNAL CANNABIS USE DURING PREGNANCY AND RISK OF PSYCHOTIC SYMPTOMS IN THE OFFSPRING

**DOI:** 10.1093/schbul/sby017.564

**Published:** 2018-04-01

**Authors:** Koen Bolhuis, Steven A Kushner, Manon H J Hillegers, Henning Tiemeier, Hanan El Marroun

**Affiliations:** 1 Erasmus Medical Center; 2 Erasmus Medical Center, University Medical Center Utrecht

## Abstract

**Background:**

Cannabis use has repeatedly been associated with psychotic symptoms, with persistent risks beyond the direct effects of exogenous cannabinoids. However, it remains unknown whether cannabis use during pregnancy is a causal risk factor for psychotic symptoms in the offspring, or whether this relationship is explained by shared etiological factors, such as genetic and environmental vulnerabilities. More innovative study designs are needed to address this question. Here, we examined the adverse effects of cannabis exposure during pregnancy on psychotic symptoms in pre-adolescent offspring. Such a method would help causal inference as comparisons can be made between the observed associations of maternal versus paternal cannabis use during pregnancy and the risk of psychotic symptoms in the offspring. If the association between cannabis use and psychotic symptoms is causal, early intra-uterine exposure to cannabis could potentially affect neurodevelopment and, hence, contribute to the pathogenesis of psychotic phenomena in children who have not yet used cannabis themselves.

**Methods:**

This study used data from the Generation R Study, a prospective population-based birth cohort from Rotterdam, the Netherlands. Participants were included if data on maternal cannabis use during pregnancy of offspring psychotic-like symptoms at age ten years were available (N = 3692). To determine cannabis exposure, we used prospective maternal self-reports during pregnancy and cannabis metabolite levels from urine. Paternal cannabis use during pregnancy was obtained through maternal report. At age ten years, children were queried regarding psychotic symptoms. Ordinal logistic regression was conducted to investigate whether maternal and paternal cannabis use were associated with offspring psychotic symptoms. In a secondary analysis, a distinction was made between maternal cannabis use exclusively before versus continued maternal cannabis use during pregnancy. All models were adjusted for covariates that were previously associated with cannabis use in this cohort.

**Results:**

Maternal cannabis use was associated with an increased risk for psychotic symptoms in their offspring (n = 183, ORadjusted=1.38 [95% CI 1.03–1.85]). Estimates were comparable for cannabis use exclusively before pregnancy versus continued cannabis during pregnancy (cannabis use before pregnancy: n = 98, ORadjusted=1.39 [95% CI 0.94–2.06]; continued cannabis use during pregnancy: n = 85, ORadjusted=1.37 [95% CI 0.90–2.08]). Paternal cannabis use was significantly associated with offspring psychotic symptoms (n = 297, ORadjusted=1.44 [95% CI 1.14–1.82]).

**Discussion:**

Using data from a large population-based birth cohort, we demonstrated that maternal and paternal cannabis use were each associated with offspring psychotic symptoms at age ten years, well before the risk period of adolescent cannabis use initiation. Notably, estimates were similar for maternal cannabis use exclusively before pregnancy versus continued cannabis use during pregnancy. Moreover, estimates were comparable for maternal versus paternal cannabis use during pregnancy. This suggests that common etiologies, rather than solely causal intra-uterine mechanisms, underlie the association between parental cannabis use and offspring psychotic symptoms, shedding potential new light on the debated causal path from cannabis use to psychosis. Our findings indicate that diagnostic screening and preventative measures need to be adapted for young people at risk for severe mental illness, and that these programs need to offer a family-focused approach.

